# Premature ovarian failure in a woman with a balanced 15;21 translocation: a case report

**DOI:** 10.1186/1752-1947-5-250

**Published:** 2011-06-29

**Authors:** Sayedehafagh Hosseini, Marzieh Vahid Dastjerdi, Zahra Asgari, Haydeh Samiee

**Affiliations:** 1 Arash University Hospital, Department of Obstetrics & Gynecology, Tehran University of Medical Sciences, Tehran, Iran

## Abstract

**Introduction:**

A case of premature ovarian failure with concomitant findings of Robertsonian translocation between 15 and 21 chromosomes is reported here. The aforementioned karyotypic aberration has not been reported in the context of premature ovarian failure to date.

**Case presentation:**

We present a case of premature ovarian failure in a 27-year-old infertile Kurdish Iranian woman with a Robertsonian 15;21 translocation.

**Conclusions:**

The diagnosis of premature ovarian failure of unknown etiology, but with karyotypic evidence of a balanced autosomal translocation, suggests the possible role of autosomal genes in the pathogenesis of ovarian follicular attrition.

## Introduction

A significant family history of early menopause is found in about 5% of cases of premature ovarian failure (POF) [1]. To determine the underlying basis of POF, genetic causes with a range of proposed loci are currently under investigation. Out of every 900 babies, one is born with a Robertsonian translocation (cited for the first time in 1964 by Gustavsson and Ingemar), showing that this translocation is the most common, significant and recurrent structural rearrangement known in man.

## Case presentation

Our patient, a 27-year-old Iranian Kurdish woman under evaluation for infertility, had experienced secondary amenorrhea from the age of 24. She had received hormonal replacement for the past three years, which resulted in cyclical bleeding, but she remained anovulatory. The Karyotype of the proband showed a translocation between chromosomes 21 and 15:45,XX,t (21;15).

She had regular menstruation cycles from the age of 13 until 21 years of age. Her height and weight were in the 90th and 50th percentiles, respectively, and she had a body mass index of 21 kg/m^2^. Her arm span to height and upper to lower segment ratios were both normal. With regard to her pubertal status, she was Tanner V for pubic hair and Tanner IV for breast growth. Her genitalia were normal and she had no virilized or dysmorphic features. Her intellectual capacity was in the normal range and she had a full-time career as a teacher.

No positive family history was noted regarding premature menopause, infertility and subfertility, smoking, chemotherapy, radiation or autoimmune diseases. The results of cytogenetic and molecular studies using polymerase chain reaction (PCR) techniques for fragile × mutations or premutations were negative.

Serum anti-thyroid, anti-ovarian and anti-adrenal antibodies were absent. Her estradiol level was 32 pg/mL and serum anti-mullerian hormone was 0.34 μg/L. She denied any history of pelvic inflammatory or sexually transmitted diseases. Additionally, no sign of pelvic surgery was observed. An ultrasound examination of the pelvis revealed a normal uterus measuring 68 × 29 mm, and the right and left ovaries were 24 × 20 mm and 23 × 21 mm, respectively. One selectable antral follicle (4.6 mm) was also seen.

A hysterosalpingogram (performed at the infertility center's routine request) confirmed normal uterine and tubal anatomy. Hormonal evaluation showed elevated follicle stimulating hormone (FSH) (25IU/mL) and leutenizing hormone (LH) (22IU/mL) levels. Her thyroid stimulating hormone (TSH), testosterone and prolactin levels were within normal limits.

## Discussion

Premature ovarian failure is a common occurence in the context of balanced X: autosomal translocations. Chromosomal imbalance can increase oocyte atresia, because after meiosis is initiated, × inactivation is not operative in germ cells [2].

It is possible that translocations such as X monosomy (Turner syndrome) lead to premature ovarian failure by causing aberrations in pairing or X inactivation during folliculogenesis [2] rather than interrupting specific genes that are important in ovarian development.

The most common Robertsonian translocations apparently have the same breakpoints and arise mainly during oogenesis, predominantly during meiosis [3]. During chromosomal pairing and condensation, failure at checkpoints (specific locations along chromosomes) provokes germ cell death. Chromosome dynamics may be sensitive to structural changes, while modification by translocations might provoke apoptosis at meiotic checkpoints [2]. Robertsonian translocation between chromosomes 13 and 14 has recently been reported in a 19-year-old Japanese woman with secondary amenorrhea [4].

There are four autosomal translocations in women with premature ovarian failure: 46 XX,t (2;11), 45,XX,t (13;14) [4], 46,XX,t (2;15) [1,5], and mosaicism 45,XX,ROB (13;21)(q10;q10)/46,XX in 55% of the cells [6]. An approximately five-years earlier menopause has previously been described with trisomy 21 [7]; therefore, a critical balance of 'determinant' genes within this chromosome may influence reproductive lifespan.

## Conclusions

As trisomy 21 is described in association with reduced ovarian reserve [3], the present translocation risk for such an eventuality is particularly escalated. In addition, given the reduced ovarian reserve, although fertility prognosis with these karyotype gametes remains suboptimal this feature has an increased risk of conceiving a fetus with trisomy 15 and monosomy 21 or 15. To minimize the risk of fetal aneuploidy, donor egg *in vitro *fertilization (IVF) provides a reassuring alternative.

Based on our medical research of English and Persian language articles, there seems to be no previously published report identifying a Robertsonian translocation between 15 and 21 chromosomes accompanied by either early menopause or reduced ovarian reserve. This finding merits widespread exploration to find whether 15;21 translocation results in disruption of ovarian folliculogenesis or follicular atresia and an early decline in ovarian follicles. Some aspects of this case will be clarified after the Human Genome Project is complete.

## Consent

Written informed consent was obtained from the patient for publication of this case report and any accompanying images. A copy of the written consent is available for review by the Editor-in-Chief of this journal.

## Competing interests

The authors declare that they have no competing interests. The authors have fulfilled all conditions required for authorship. The authors have no previous publications similar to this study.

## Authors' contributions

All authors analyzed and interpreted our patient's data. The first author was the major contributor to writing the manuscript. All authors read and approved the final manuscript.
